# The Origin of Spectacles

**Published:** 1899-02

**Authors:** 


### The Origin of Spectacles.

Dr. E. P. Daviss (*South Western Med. Record*, August) says that it is to Charles II., of England, that the world owes the discovery of lenses as an aid to vision. After his escape to France, and after his father was beheaded, he became a pensioner of the great Louis XIV., and while living his indolent and dissolute life in Cologne he met an expert artisan in glass, and, accidentally looking through a small lens belonging to this workman, he found that it greatly benefitted his very imperfect vision. He at once bought *all* his lenses and took the artisan into his own employ.

The young prince, born with myopic astigmatism (irregular near sight), by mathematical calculation based upon these crude lenses, thus accidentally discovered perfection in the glasses he is said to have worn, and by which his vision was raised from one-half of normal sight up to perfect vision. These glasses are now in the British Museum and were the first spectacles ever made for visual purposes.

When Charles was called to England and crowned Charles II., he took his French artisan and about twenty others with him, and it is said that as many as six thousand lenses were made before he got what he wished, and what finally gave him perfect vision. These glasses, when perfected, had cost the prince over £100,000.

The good, accidentally it would seem, that followed Charles' exile to France has been worth many times over to mankind the loss of his father's head, the fear of the same fate having been his incentive to flight. The Duke of Monmouth, Charles II.'s favorite son, also near-sighted, had but one eye, hence the origin of the "monocle." His father's artisans improved his vision with a single lens worn over his better eye.—*N. Y. Med. Journal.*

